# Digital Health Applications in Access to Health Service From the Perspective of Older Adults: “It Is Difficult to Keep up With the Digital Age …”

**DOI:** 10.1111/phn.70005

**Published:** 2025-07-14

**Authors:** Damla Şahin Büyük, Çetinkaya Aynur, Bilgin Nurcan

**Affiliations:** ^1^ Department of Public Health Nursing Faculty of Health Science Manisa Celal Bayar University Manisa Turkey; ^2^ Department of Nursing Management Faculty of Health Science Manisa Celal Bayar University Manisa Turkey

**Keywords:** digital age, digital health, digital health experience, digital health information systems, digital health perception, digital health systems, older adults

## Abstract

**Objective:**

This study aims to explore the experiences of older adults in Türkiye regarding widely used digital health information systems in healthcare and to develop a conceptual process model based on these experiences.

**Design and Sample:**

The research was conducted based on a qualitative approach using a grounded theory design. In‐depth interviews were conducted with nine individuals aged 65 and above, selected through criterion and snowball sampling. Data were analyzed using content analysis.

**Results:**

The categories identified through in‐vivo coding were: “*We have grown old…*” (*f* = 115), “*It is necessary to be prepared for everything*” (*f* = 69), “*It is difficult to keep up with the digital age*” (*f* = 97), and “*It is not difficult*” (*f* = 93).

**Conclusion:**

The study revealed that older adults prefer traditional methods for healthcare access and encounter barriers like lack of knowledge, usability issues, and technological anxiety with digital systems. However, they also recognized the benefits of digital health. It concludes that digital services should be more inclusive and user‐friendly for older adults.

## 1 Introduction

Technological advancements and the widespread use of the internet and mobile devices have rapidly transformed the way health services are delivered and accessed globally (Frishammar et al. 2023; Mercan et al. 2020). This transformation has been particularly influential in driving digitalization and the expansion of mobile healthcare services within the healthcare sector. Digital health, or digital healthcare, is a broad, multidisciplinary concept including ideas at the intersection of technology and healthcare (World Bank 2023). The digital health framework covers various systems such as mobile health (mHealth) applications, electronic health records (EHRs), electronic medical records (EMRs), telehealth, and telemedicine (Wilson et al. 2021). While the scope of digital health services is extensive, their implementation can vary depending on factors such as the technological infrastructure available in different countries, the level of adoption by users, and the overall readiness of the system (World Bank 2023).

One of the key factors influencing global trends in the development of digital health applications is the growing number of older adults. As their population increases, so does the prevalence of chronic diseases, placing a greater burden on healthcare systems. To manage this rising demand, it becomes essential to increase both the usage rate and the variety of digital health systems (Urban 2017; World Bank 2023). The literature identifies the most significant barriers older adults face in accessing digital health services as limited digital literacy, cognitive and sensory impairments, physical limitations, concerns about privacy and security, lack of social support, and reluctance to adopt new technologies (Barnard et al. 2013; Ilgar and Bilgili 2023; Kırca et al. 2021; Money et al. 2024; Urban 2017; Vainieri et al. 2023). Digital health systems offer advantages for older adults who face challenges in accessing healthcare, such as enabling access to certain services remotely and reducing waiting times. However, for older adults to benefit from these advantages, they must possess digital literacy and proficiency in using digital devices (Almulhem 2023; Ilgar and Bilgili 2023). Nevertheless, it is well‐known that older adults generally have lower levels of both literacy and digital skills compared to younger people. This gap is attributed to factors such as age‐related declines in sensory and cognitive abilities, feelings of inadequacy when using technological tools, lack of motivation, and various psychosocial barriers, design‐related obstacles such as small screens, complex interfaces, and inadequate usage instructions (Barnard et al. 2013; Ilgar and Bilgili 2023; Kırca et al. 2021; Money et al. 2024). In addition to age‐related factors, socioeconomic status, gender, and education level play significant roles in shaping older adults’ access to digital health services. Limited income and education can restrict access to technology and digital literacy, while gender inequalities often lead to older women facing more disadvantages compared to older men (Chen et al. 2024; Shi et al. 2024; Vainieri et al. 2023).

This situation causes older individuals to face greater difficulties in adapting to digital processes and acquiring digital skills, making them more dependent on support and ultimately leading to digital exclusion (Sieck et al. 2021). In today's rapidly digitalizing world, the concept of digital exclusion—related to access, ability to use digital systems, and financial affordability—is viewed as a “super” social determinant of health (Gallistl et al. 2021; Lu et al. 2022; Sieck et al. 2021). While factors like low income, disability, and living in rural areas are recognized as significant risk factors for digital exclusion, the literature highlights that age is the most important risk factor (Money et al. 2024). This also brings attention to ageism in the digital realm. Ageism is a significant barrier affecting the design, adoption, and use of digital technology (Kutsal 2023).

In Türkiye, internet access (95.5%) is relatively high compared to the global average (63%) indicating that the necessary resources for utilizing digital health applications are available to citizens (Ritchie et al. 2023; TurkStat 2023). However, despite these resources, only 40.7% of older adults in Türkiye use the internet (TurkStat 2023). Furthermore, Türkiye ranks last among European countries and 115th globally in terms of digital skills, with this issue being even more pronounced among older adults (We Are Social 2022). When the increasing health problems of older adults and their digital exclusion are considered together, it becomes clear that, without proper support mechanisms, older adults —who are the most in need of health services—could be excluded from the benefits of digital health applications (Almulhem 2023).

Although studies on older adults’ use of digital systems in general exist in the literature, those specifically focusing on their use of digital health services remain limited (Almulhem 2023; Cho et al. 2008; Frishammar et al. 2023; Ilgar and Bilgili 2023). Most studies conducted in Türkiye either focus on the general population or examine older adults’ experiences with digital technologies outside the context of healthcare. However, in the field of health, research specifically addressing how older adults use digital health applications remains notably limited (Ilgar and Bilgili 2023; Mercan et al. 2020). This creates a gap in understanding the challenges, perceptions, and needs of older individuals when accessing digital health services. As healthcare becomes increasingly digital and the older population continues to grow, it is important to understand how older adults perceive and use digital platforms in order to support equal access to care and prevent digital exclusion. To fill this gap, this study explores the perceptions and experiences of older adults in Türkiye regarding two widely used national digital health information systems—e‐Pulse and the Central Physician Appointment System (CPAS)—specifically focusing on their access to healthcare services.

By utilizing a qualitative design, this study allows for an in‐depth exploration of older adults' perceptions and experiences with digital health applications. From a public health nursing perspective, this approach is critical for understanding the barriers to older adults' access to digital health systems and the psychosocial factors at play. Public health nurses can leverage these findings to create more targeted, individualized interventions aimed at improving older adults' access to digital health services. In doing so, they can play a significant role in ensuring equal access to healthcare, particularly for older adults at risk of digital exclusion.

## 2 Methods

### 2.1 Study Design and Setting

The research was conducted using a qualitative approach with a grounded theory design, aiming to interpret human experience in its natural context. The most significant contribution of the qualitative research design is examining the subject of research from the perspectives of the individuals involved and revealing the underlying social structures and processes (Polit and Beck 2020). Grounded theory is an approach that facilitates a profound understanding of experiences and fosters the creation of new theories derived from them. This method is rooted in the data itself, allowing theories to naturally emerge during the process of data analysis (Corbin and Strauss 2014). Because nurses deliver comprehensive care to patients, nursing research ought to encompass the entirety of human experiences related to a given topic. This design was chosen for this study because it provides an excellent framework for understanding the experiences and behaviors of the participants.

This study was conducted in Manisa, a province located in western Türkiye, which has a mixed urban and rural population structure, providing a suitable context to explore older adults' experiences regarding the use of digital health systems. The data were collected through in‐depth interviews between July and September 2024.

### 2.2 Participants

Theoretical sampling was used to ensure that data collection was guided by emerging concepts rather than a pre‐determined sampling plan in the study. Interviews were conducted with nine older adults (seven males, two females), selected using the purposive sampling method. To collect initial data criteria in this research criteria were as follows: Living in Manisa city center, being over 65 years of age, having primary school education or above, having smartphone/computer/internet access and volunteering to participate in the research. Interviews were conducted with the participants identified through purposive sampling, and the number of participants was determined by theoretical (data) saturation. In qualitative research, theoretical saturation is used as the sampling criterion. This means that saturation is reached when no new code emerges during interviews, and researchers can terminate the interview process and no longer need to increase the sample size (Guest et al. 2006). Thus, in the current research, data collection was continued through interviews until saturation was reached. Saturation was achieved after interviewing the 7th participant, and two additional participants were interviewed before concluding the study (*n* = 9).

The descriptive characteristics of the participants are outlined in Table 1. The participants’ ages ranged from 65 to 77. Most of the participants were male and married, while eight reported an average economic status. Five participants had completed high school. All participants had access to a smartphone, computer, or the internet. Eight participants reported using the e‐Pulse system, and six were using the CPAS. None of the participants were using telehealth services, and only three were able to navigate digital services independently. The skill scores for using digital healthcare applications among participants ranged from 3 to 10 (Table 1).

**TABLE 1:** Descriptive characteristics of the sample.

Participant no.	Age	Gender	Economic status	Marital status	Education level	Smartphone/computer/internet access	Using e‐Pulse	Using CPAS	Using tele health	Using digital services on your own	Perception of competence in using digital health service applications^a^
P1	65	F	Moderate	Married	Primary school	Yes	Yes	Yes	No	Yes	6
P2	71	M	Moderate	Married	Primary school	Yes	Yes	Yes	No	Yes	8
P3	71	M	High	Married	High school	Yes	Yes	No	No	No	7
P4	67	F	Moderate	Single	High school	Yes	No	No	No	No	4
P5	66	M	Moderate	Married	Secondary school	Yes	Yes	Yes	No	No	6
P6	72	M	Moderate	Married	High school	Yes	Yes	No	No	No	3
P7	75	M	Moderate	Single	Primary school	Yes	No	No	No	No	4
P8	77	M	Moderate	Married	High school	Yes	Yes	Yes	No	No	5
P9	67	M	Moderate	Married	High school	Yes	Yes	Yes	No	Yes	10

^a^ Shows the self‐perceptions of older adults on a 10‐point scale.

### 2.3 Data Collection Tools

In this study, data collection involved a descriptive form capturing the socioeconomic profiles of participants, alongside a semi‐structured interview guide featuring open‐ended questions designed based on insights from the literature.

*Socio‐Demographic Information Form*: This form, developed by the researchers based on the existing literature, contains 16 questions on variables such as age, gender, marital status, educational background, economic condition, longest place of residence, use of digital health services, and perceived competence in using digital health applications. The perception of competence in using digital healthcare applications was assessed through a single self‐reported question, using a 10‐point scale. Participants were asked to assign a score between 1 and 10, where 10 represented the highest level of competence, in response to the question: “If you were to rate your ability to use digital health applications on a scale of 1 to 10, how many points would you give yourself?” (with 10 indicating the highest level of competence) This question was presented in the form of a 10 cm visual scale for participants to indicate their response.

*Semi‐structured Interview Form*: The interview form consists of 10 questions developed by the researchers based on a review of the literature and aligned with the research objectives (Almulhem 2023; Barnard et al. 2013; Frishammar et al. 2023; Gallistl et al. 2021; Henchoz et al. 2008; Kaihlanen et al. 2022; Lu et al. 2022; Money et al. 2024). The purpose of the form is to assess older adults’ use of healthcare services, their perceptions of digital health applications, and their experiences with these applications, and to evaluate their digital health skills and adaptation. The semi‐structured interview form was reviewed by two experts in Public Health Nursing and one expert in Family Medicine, who provided feedback and suggestions for improvement. Following this expert review, a pilot study was conducted with three older adults to ensure the clarity and functionality of the questions. Based on the pilot results, an interview question was removed and two interview questions were revised that due to repetition, ambiguity, or limited relevance to the research objectives. These modifications improved the clarity and flow of the interviews, ensuring the questions effectively captured participants' experiences. The interview form was then finalized. The data obtained during the pilot study were excluded from the final analysis. Sample questions from the interview form include: *“What are your thoughts on visiting a doctor or health institution when your health deteriorates (when you feel sick or uncomfortable)?”, “Do you use digital health applications? Which ones, and what do you know about them?”, and “Can you describe the challenges and ease of using digital health applications?”*

### 2.4 Data Collection Procedure

In this qualitative study, prior to the in‐depth interviews, participants were informed about the study's purpose, scope, and the intended use of the data. The participants were asked to provide informed consent for the interviews to be audio‐recorded and for their responses to be used as anonymous quotations. They were also informed of their right to withdraw from the study at any point without providing a reason, and only individuals who consented voluntarily were included in the study.

After completing the socio‐demographic information form, face‐to‐face interviews were conducted using a semi‐structured interview guide. The interviews were recorded using a digital voice recorder, which ensured the collection of complete and accurate responses (Olympus digital voice recorder VN‐8500 PC; 200154018). The primary researcher (DŞB) conducted interviews with new participants until data saturation was reached. Interviews were held in a quiet setting preferred by the participants (e.g., at their homes or in a meeting room at a pension association) to avoid interruptions. The interviews were conducted in Turkish and lasted approximately 25–35 min.

### 2.5 Data Analysis

The data were analyzed using NVIVO 12 Pro software. Initially, a pre‐reading of the interview texts (in oral form) was conducted to familiarize with the data and identify preliminary information. Open coding was used to label concepts and categorize them according to their attributes and dimensions to analyze the participants’ responses. An open coding list was generated through qualitative data analysis (number of codes: 31). Then, similar codes were collected into sub‐categories. For certain categories, an in vivo coding process was implemented within the context of the qualitative research methodology of grounded theory. This methodology involves the generation of a theme label directly from the data by utilizing participants’ own expressions (Zamawe 2015). The analysis process was followed by selective coding, and the conceptual process of the participants’ use of the health information systems in healthcare experiences was defined with the identified focal categories. An inductive approach was employed in this study, thereby allowing for the identification of themes inherent in the data (Bengtsson 2016). The results were presented using citation/frequency abbreviations, where “f” indicated the number of citations, and “P” was used for participant numbers. Additionally, since the interviews were conducted in Turkish, the transcripts were translated into English by an expert fluent in both Turkish and English.

### 2.6 Validity and Reliability in Research

The research team comprised two public health nurses and one expert in nursing administration. Two researchers (A.Ç. and D.Ş.B.) were certified in qualitative data analysis. Throughout the interviews and data analysis, the authors prioritized honesty, integrity, and fostering an environment for self‐expression. To ensure trustworthiness, Lincoln and Guba's (1985) criteria were applied. Credibility was established through prolonged engagement with data and investigator triangulation, whereby multiple researchers independently analyzed the data until consensus was reached (Carter et al. 2014). Dependability was achieved by thoroughly documenting each stage of data collection and analysis. Confirmability was supported by detailed field notes. Transferability was ensured by providing comprehensive descriptions of participants and the research setting, allowing readers to assess the applicability of the findings to similar contexts. The study report adhered to the COREQ checklist, which is designed to promote transparent and comprehensive reporting in qualitative research, particularly in the health field (Tong et al. 2007).

### 2.7 Ethical Considerations

Ethical approval for the study was obtained from the ethics committee of a university (Decision date: 10.07.2024; Decision No: 20.478.486/2508). Informed written consent was obtained from all participants. To protect the privacy of participants, no identifying information was included in the research report. Only volunteers were included in the study.

## 3 Results

The study involved nine older adults (7 males, 2 females) aged 65–77 years, mostly married and with moderate economic status. Education levels varied from primary to high school. All participants had smartphone/internet access; most used e‐Pulse (*n* = 7) and CPAS (*n* = 6), but none used telehealth. Three participants reported independently using digital health services. Participants’ self‐perceived competence in using digital health applications ranged from 3 to 10 on a 10‐point scale (Table 1).

Assuming that human experience unfolds across emotional, cognitive, and behavioral dimensions (contextual explanations), this grounded theory study used constant comparative analysis to trace a sequential conceptual process that moves from perceptions of aging to engagement with digital health technologies.

Within the emotional dimension, the category *“We have grown old…”* (*f*  =  115) captures how chronic illnesses, somatic complaints, and prior encounters with healthcare shape participants’ sense of growing older; this emotional foundation guides all subsequent appraisals. In the cognitive dimension, the category *“It is necessary to be prepared for everything”* (*f*  =  69) describes how older adults evaluate risk, apply an initial decision filter (traditional remedies vs. rational medication use), and choose when to seek professional care—either delaying or acting urgently. The behavioral dimension then splits into two contrasting categories that emerge after contact with digital health systems: *“It is difficult to keep up with the digital age”* (*f*  =  97), marked by unfamiliarity, fear of error, and a need for training, and *“It is not difficult”* (*f*  =  93), characterized by ease of use, mobile convenience, and time savings.

**FIGURE 1:**
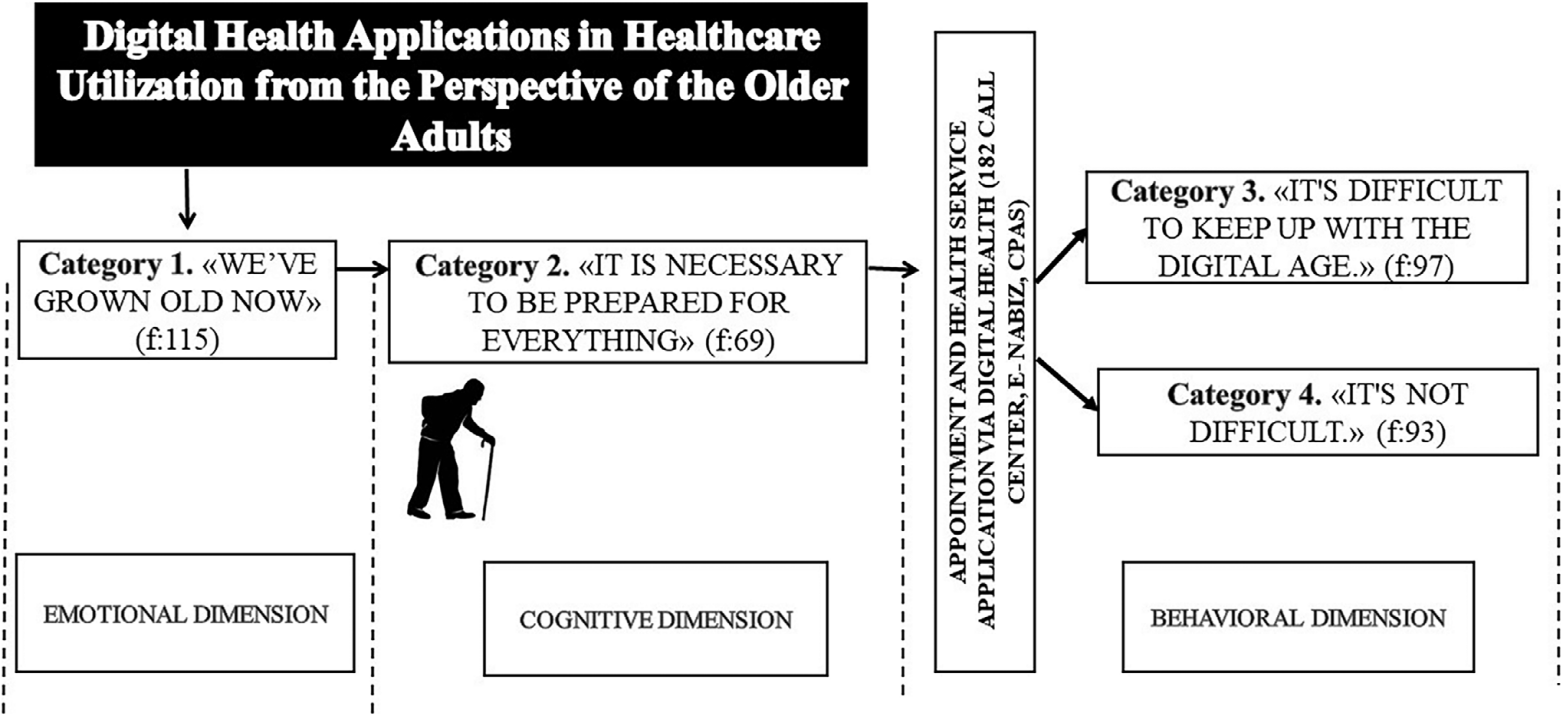
Conceptual process model of older adults’ digital‑health experiences in health‑care utilization: related categories and frequency counts.

Thus, emotional perceptions frame the aging experience, those perceptions inform cognitive evaluations, and cognitive evaluations translate into concrete behavioral engagement—or avoidance—with digital health applications. The three dimensions are linked through the subcategories listed with detailed codes and frequencies in Table 2, providing a comprehensive account of the barriers and facilitators older adults encounter in digital health use. A schematic overview of the model is presented in Figure 1.

**TABLE 2:** Codes and categories derived from the experiences of older adults in Türkiye with widely used digital health information systems (*n* = 9).

Dimension: Contextual explanation	Category	Sub‐category	Codes (open coding)
Emotional: The meaning of aging	Category 1:“We have grown old…” (*f* = 115)	1. Diseases (*f* = 41)	1. Chronic diseases (*f* = 36)
			2. Age‐associated cognitive decline (*f* = 5)
		2. Utilized health care unit (*f* = 26)	1. Family health center (*f* = 12)
			2. Hospital (*f* = 8)
			3. Emergency department visit (*f* = 6)
		3. Somatic complaints (*f* = 22)	1. Pain (*f* = 15)
			2. Cough (*f* = 7)
		4. Medications (*f* = 13)	1. Medication use (*f* = 5)
			2. Prescription renewal (*f* = 8)
		5. Gratitude perception (*f* = 13)	1. Spiritual gratitude (*f* = 9)
			2. Thankfulness for health improvements (*f* = 4)
Cognitive: Healthcare service decision‐making process	Category 2: “It is necessary to be prepared for everything” (*f* = 69)	6. First decision filter (*f* = 41)	1. Traditional home remedies (f = 28)
			2. Rational drug practice (*f* = 13)
		7. Reluctant, delayed healtcare‐seeking (*f* = 14).	1. Only when I get realy bad (*f* = 8).
			2. Reluctantly (*f* = 6).
		8. Immediate healthcare seeking in illness (*f* = 14)	1. Immediate direct application (*f* = 8)
			2. If I am really sick, it is urgent (*f* = 6)
Behavioral: Forms of responding	**Category 3**: “It is difficult to keep up with the digital age” (*f* = 97)	9. “I don't know, but I can do it if I learn” (*f* = 47)	1. Perceived need for training (*f* = 26)
			2. Preference for step‐by‐step guidance (*f* = 11)
			3. Perceived learnability (*f* = 10)
		10. “I feel uneasy, it would be easy if it were…” (*f* = 11)	1. Fear of doing something wrong (*f* = 7)
			2. Desire for age‐friendly design (*f* = 4)
		11. Perceived difficulty & complexity (*f* = 39)	1. It is difficult (*f* = 31)
			2. It is troublesome, complicated, multi‐stage, challenging (*f* = 6)
			3. It is tedious (*f* = 2)
	**Category 4**: “It is not difficult” (*f* = 93)	12. Positive usability & satisfaction (*f* = 48)	1. Easy, comfortable (*f* = 32)
			2. Satisfied (*f* = 16)
		13. Mobile convenience (*f* = 28)	1. Everything is on the phone (*f* = 16)
			2. Very useful (*f* = 12)
		14. Time efficiency (*f* = 17)	1. No waiting in line (*f* = 11)
			2. Quick hospital appointment booking (*f* = 6)

The terms “difficult” and “difficulty” were mentioned 36 times by 9 older adults and were selected as category labels through in‐vivo coding. Additionally, the term “distress” was mentioned 33 times by the participants. Participant 9's statement, “It is difficult to keep up with the digital age,” was chosen as the title because it effectively summarizes the challenges older adults face in using digital health applications and connects to other relevant categories.

Below, each category is presented in sequence and illustrated with representative participant quotations:

*Category 1: “We have grown old…” (f = 115)*

This category, which received the highest number of citations among nine older adults, was derived through in‐vivo coding from Participant 1's statement. Participant 1, a 61‐year‐old woman with a primary school education, shared, *“We have grown old. I have diabetes, high blood pressure, and cholesterol; I use a lot of medication.”* Her remarks illustrate her perception of aging. The phrase “We have grown old” can be interpreted as an emotion reflecting the older adults’ acceptance of the effects of aging and the challenges they face in adapting to evolving physical, social, and technological processes over time. This category encompasses a description of aging, including effects such as a decline in physical capacity, a slowing of cognitive functions, a deterioration in sensory abilities, and difficulties in adapting to technological advancements. This category accounted for 30.7% of all citations and includes topics such as chronic diseases, medications, health‐related complaints, and experiences with health institutions. Additionally, participants expressed a sense of gratitude, often stating they felt “very thankful.” The sub‐category and their citation counts are as follows: Diseases (*f* = 41), Utilized health care unit (*f* = 26), Somatic complaints (*f* = 22), Medications (*f* = 13), and Gratitude perception (*f* = 13). Below are quotes from some participants related to this category:

“I have two problems now: I have a problem with my knees, and I have a herniated disc. In 2005, I had surgery for a herniated disc at L4‐L5. Now, I have problems with L2 and L3 again. … Other than these two problems, thank God, I have no other problems.” P2, 71 years old, Male, Primary school graduate.

“My wife prefers traditional, non‐medical treatments. She watches them on the phone and TV more. I say, ‘Let's go to a doctor first. If that doesn't work, then we can try them.’ But I don't find them very useful, and I'm not in favor of them. I only take prescribed medications from the pharmacy. I don't use medicines, herbs, or anything the neighbor suggests is good for me. I am very careful about that.” P3, 71 years old, Male, High school graduate.

“The last time I went to the health center was two weeks ago. I have prostate issues, so I needed to get my regular medication prescribed. Besides that, I also got a flu medicine in case I catch the flu and painkillers in case I have any pain.” P5, 66 years old, Male, Secondary school graduate.

Category 2: “It is necessary to be prepared for everything” (*f* = 69)

This category, accounting for 18.4% of all attributions from the participants, reflects the participants’ views on the decision‐making process for seeking health services. From the perspective of older adults, “It is necessary to be prepared for everything” can be interpreted as a reflection of the life‐long experiences gained and the adaptive capacity developed to cope with uncertainties. This phrase highlights the need for older adults to be prepared for the physical, social, and psychological changes encountered during the aging process and illustrates the resilience mechanisms they have cultivated in response to these challenges. Many older adults initially relied on traditional home remedies and only sought professional healthcare after a delay, often following the use of rational drug applications. However, some of them visited health institutions directly. The sub‐categories and their citation counts are as follows: “First decision filter” (*f* = 41), “Reluctant, delayed healtcare‐seeking” (*f* = 14), “Immediate healthcare seeking in illness” (*f* = 14).

Participant 1 described how she only seeks professional medical help when her condition worsens:

“If my health deteriorates, I first try to do something myself at home, but if I get worse, of course, I go to the hospital. But if I have a headache, for example, I don't take pills immediately. If my blood pressure rises, I drink lemon juice, but that is very rare. I don't know anything else. I don't need to rely on what I hear from others because there are doctors.” P1, 65 years old, Female, Primary school graduate.

The expressions “if I have to” and “if I get worse” reflect how older adults often experience the healthcare application and appointment process reluctantly, only seeking help when they feel it's necessary. Below are additional participant quotes that highlight this category:

“I had to go to the hospital immediately. I mean, for urgency, I go to the doctor without waiting. But for minor issues, I know myself and I already know what to do. I don't go immediately. For non‐urgent conditions, I make an appointment and go to the doctor.” P2, 71 years old, Male, Primary school graduate.

“If I am very sick, I will call 112, but if it is a normal illness, I do nothing. I will lie down and rest for a while, then go on my way. If it is not urgent, I do not go to the hospital.” P4, 67 years old, Female, High school graduate.

*Category 3: “It is difficult to keep up with the digital age” (f = 97)*

This category highlights the challenges older adults face with the digital processes involved in making health service appointments and accessing services through platforms (Alo 182, e‐Pulse, and CPAS.) It received ¼ (%25.9) of all citations and was derived from Participant 9's statement using in‐vivo coding. Older adults may find modern systems (e.g., smartphones, computers, online applications) hard to understand, feel overwhelmed by complex interfaces, learning new terminology, or keeping up with constantly changing digital trends, and experience a sense of falling behind due to generational gaps. Three sub‐categories were identified within this category, each highlighting different aspects of the difficulty: “I don't know, but I can do it if I learn” (*f* = 47), “Perceived difficulty and complexity” (*f* = 39), “I feel uneasy, it would be easy if it were…” (*f* = 11). Below are some participant quotes related to this category:

“If I had a very serious illness, I would think of going to the doctor, even if I don't like it much. I make the appointment by calling (182). I call, but I don't make the appointment myself; either my son, daughter, or granddaughter does it. Not because it's more convenient, but because I don't want to deal with it. I don't know how to, and I don't like it. At this age, it's hard for us to learn. If there were someone or somewhere to teach this, of course, I'd like to learn.” P5, 66 years old, Male, Secondary school graduate.

“I don't know e‐Pulse, but I would like to use it. If there were a trainer, we could have learned it by practicing. Many things could be tracked there, and it would be very convenient for us. The problem is that it requires technical skills, and I don't have much practice in this area. From our perspective, we can't speed up these processes. Since we don't use computers much, we definitely need support at this age.” P5, 66 years old, Male, Secondary school graduate.

“As a matter of fact, it is difficult, yes, we can call it that—it is difficult. I'm afraid of dealing with it because I worry that I might do something wrong, like making a mistake during the process. It's difficult to make an appointment, and I don't bother looking at the other options anyway. We don't use them. These processes should be shorter; they confuse us.” P8, 77 years old, Male, High school graduate.

“It would be good if it were easier because when making an appointment, you first have to enter the province, then write the city, choose the clinic, select a doctor, and so on—it's a hassle. You have to enter a lot of information and click through many things… It's very tedious.” P1, 65 years old, Female, Primary school graduate.

*Category 4: “It is not difficult” (f = 93)*

This category focuses on the experiences of older adults who did not find it difficult to use digital health applications (Alo 182, e‐Pulse, and CPAS) for making appointments and accessing health services. Older adults' description of digital health services as “not difficult” reflects their growing selfconfidence, successful adaptation, and—in some cases—the support they receive to use these systems effectively. This finding shows that they have adopted a more positive attitude toward digital health technologies and are motivated to use them efficiently. It accounted for ¼ (24.8%) of all citations and was developed through in‐vivo coding. Participant 2, a 71‐year‐old male, emphasized his ease with the system, stating three times, *“I do not have any difficulty in using this system. It is not difficult for me, so there is nothing difficult for me.”* The sub‐category and citation counts are as follows: “Positive usability & satisfaction” (*f* = 48) “Mobile convenience” (*f* = 28), and ‘’Time efficiency (*f* = 17). Below are some participant quotes related to this category:

“When I get sick, of course I feel bad, and if I am really very sick, I make an appointment for my doctor. No one helps me make an appointment; I can do it by myself. My son taught me how to make an appointment, and it's better to know it. I don't ask my son to do it for me. Even if I need to make an appointment for my wife and mother, I make the appointment for them. It's not good to always rely on my son. I feel better when I do it myself. It's easy once you know how to do it. For many years now, I've been able to manage things on my own. It's not difficult for me because I know how to do it. There's nothing negative about having it on a mobile phone; I think it's very good.” P1, 65 years old, Female, Primary school graduate.

“I use CPAS for appointments; I have the app on my mobile phone. I also know e‐Pulse. I use it for other things (besides appointments). I can make the appointment myself, and I'm pleased that I can handle things on my own without needing anyone else. Yes, there are difficult parts—now you have to log into e‐state and access CPAS from there, or if you go directly to CPAS, you get to the actual information after a few stages. I think it would be better for older adults to get there with fewer clicks, of course. It could be more easily accessible. The easy part is that you can avoid going to the hospital and waiting in line for a long time (laughing). Other than that, I don't know, but you save time. It's enough to be at the hospital half an hour before your appointment.” P9, 67 years old, Male, High school graduate.‘’

## 4 Discussion

In this study, we explored the experiences of older adults in Türkiye with two widely used digital health information systems and, on the basis of those experiences, developed a conceptual process model. The findings were organized within a three‐dimensional framework—emotional, cognitive, and behavioral—and synthesized into four overarching categories (see Table 2 for details). In the Discussion section, each dimension is examined in turn under separate subheadings, and the findings are interpreted in relation to the relevant literature.

### 4.1 Emotional Dimension: “We Have Grown Old”

A prominent category identified in participants’ narratives was their heightened awareness of aging and its impact on their health status and behavior. Older adults in this study frequently highlighted their chronic conditions, disease symptoms, continuous medication use, and the health institutions they visited. These statements, categorized under the category *“We have grown old,”* support the literature showing that older individuals often visit healthcare facilities due to chronic illnesses, and as age increases, so does the number of chronic diseases and hospital visits for managing these conditions (Fernández‐Olano et al. 2006). On the other hand, it is also noted that older adults with a more positive perception of their health tend to visit healthcare institutions less frequently, adopt healthier behaviors, and make greater use of preventive services (Cho et al. 2008). Consistent with the findings of this study, Henchoz et al. (2008) examined both perceived and actual health status in older adults and found that both decline with advancing age. In another study, Song and Kong (2015) explored health perceptions among older adults, revealing that perceptions of health varied: some older adults equated health with the absence of disease, while others viewed it as the presence of manageable conditions or chronic illnesses controllable through medication (Song and Kong 2015). This variability may stem from a general acceptance among some individuals of living with chronic diseases as a normative aspect of aging. In our study, while some participants predominantly highlighted the increase in health‐related challenges associated with aging, others expressed a sense of gratitude despite their health issues. This observation may align with the acceptance of aging as noted in Song and Kong's research (2015), yet it also suggests a level of satisfaction with health services and resilience in the face of adversities.

### 4.2 Cognitive Dimension: “It Is Necessary to be Prepared for Everything”

Another important category was “It is necessary to be prepared for everything,” which captured participants’ individualized and conditional decision‐making processes regarding the use of healthcare services. Within the category of *“It is necessary to be prepared for everything,”* participants in this study indicated that they preferred to rely on traditional practices rather than seek assistance from healthcare institutions for their health issues. Seeking help from healthcare institutions was often described as a last resort, only considered after self‐managed approaches failed or symptoms worsened. A limited number of respondents, however, reported that they would promptly consult a healthcare provider. While this finding appears to contradict another observation regarding the negative health perception that tends to increase with age, existing literature suggests that older adults often underestimate or normalize their health problems as they age (Henchoz et al. 2008; Spitzer and Shaikh 2022). Although this demographic acknowledges a decline in health status, they do not perceive it with the same urgency as they might have in their youth. The decision to delay or avoid formal care was shaped by a variety of reasons expressed by participants, including a belief in managing symptoms on their own (“I know myself, I don't go right away”), postponing care until symptoms worsened (“Only when it gets really bad…”), and in some cases, a preference for direct access to healthcare. These findings point not only to a cautious approach, but also to varied and personalized decision‐making processes in seeking care.

### 4.3 Behavioral Dimension: “It Is Difficult to Keep up With the Digital Age”

The category of *“It is difficult to keep up with the digital age,”* identified as a central focus of this study, highlights the significant challenges that older adults face in engaging with digital health information systems, such as e‐Pulse and CPAS. This category encompasses a wide range of individual challenges, including limited digital literacy, restricted usage practices, system complexity, fear of making mistakes, and technological anxiety. In addition, it is closely related to various psychosocial factors such as low perceived self‐efficacy, lack of motivation, social isolation, and insufficient emotional or instrumental support. In this respect, the category reveals the multidimensional nature of digital exclusion among older adults.

The most frequently reported barrier under this category was related to older adults’ digital literacy. This finding of the study is consistent with previous research indicating that limited general literacy, reduced capacity for learning new skills, and low digital literacy levels are major barriers to the effective use of digital health systems among older adults (Bertolazzi et al. 2024; Ji et al. 2024; Raihan et al. 2025; Wilson et al. 2024). Unlike the younger population, who are digital natives, older adults did not grow up in a digital environment, which places them at a disadvantage regarding digital literacy and skills, which has led to the characterization of older adults as “digital immigrants” in the literature (Kutsal 2023). Interestingly, despite their status as digital immigrants, older adults utilize health systems more frequently than younger individuals due to a higher prevalence of health issues (Song and Kong 2015). Given that one of the primary objectives of digital health services is to alleviate the burden on healthcare institutions, the inadequate engagement of older adults in these systems—stemming from their lack of knowledge and skills—contradicts this goal (World Bank 2023). Furthermore, from a rights‐based perspective, the exclusion of older adults from digital health inclusion raises concerns about the adverse effects of social determinants on this demographic, highlighting the need for targeted interventions to address these disparities.

Another significant issue raised by the participants regarding the use of digital health applications was the perceived complexity, multi‐step processes, and overall difficulty of these systems. This finding is consistent with several studies in the literature (Frishammar et al. 2023; Fox and Connolly 2018; Kırca et al. 2021; Money et al. 2024). The negative evaluations may be attributed to age‐related declines in sensory and cognitive abilities, design‐related challenges, and psychosocial barriers (Ilgar and Bilgili 2023; Kırca et al. 2021). Psychosocial factors such as low perceived self‐efficacy, lack of motivation, and limited social support may amplify the perceived complexity of digital health systems. When individuals feel unconfident in their ability to navigate technology, lack the motivation to engage, or do not have access to support networks, even relatively simple tasks may be perceived as overly complicated, burdensome, or intimidating (Hwang et al. 2025; Kırca et al. 2021). Design‐related challenges, such as small screens, complex user interfaces, and insufficient usage instructions, further contribute to the difficulties experienced by older adults when using digital health services (Barnard et al. 2013; Ilgar and Bilgili 2023; Kırca et al. 2021; Money et al. 2024; Raihan et al. 2025; Wilson et al. 2024). These findings underscore the need for the development of user‐friendly systems that promote digital inclusiveness in healthcare for older adults.

Beyond structural and usability challenges, participant statements also revealed an anxiety driven aspect of technology use. In this study, the sub‐category identified in‐vivo from a participant's statement, *“I feel uneasy, it would be better if it were…,”* highlights the fear of making mistakes or, more broadly, the technological anxiety experienced by older adults in adapting to digital health systems. In the literature, technological anxiety in older adults using digital health services is often linked to concerns about usage, such as pressing the wrong button or making incorrect transactions (Money et al. 2024; Raihan et al. 2025; Wilson et al. 2024). However, data security is also a significant factor contributing to this anxiety (Brall et al. 2019; Frishammar et al. 2023). The increasing prevalence of digital fraud may further exacerbate the fear and hesitation older adults feel when using digital health systems. This lack of trust can hinder both their initial decision to engage with these systems and their ability to continue using them over time (Frishammar et al. 2023).

### 4.4 Behavioral Dimension: “It Is Not Difficult”

The final category, “It is not difficult,” reflects the positive experiences reported by some older adults. Despite the barriers and challenges identified in the study regarding the use of digital health services, a group of participants frequently emphasized the advantages and usefulness of these systems under the category *“Not difficult.”* The findings suggest that digital health systems offer various benefits to older adults, including efficient management of health data, time and effort savings, and improved access to clinical services. Research findings indicating that older adults who have been previously exposed to technology or who receive support from their social environment tend to develop more positive perceptions of digital tools can be associated with the findings of this study (Hwang et al. 2025). However, it should be noted that these positive experiences may be mostly limited to individuals who are more familiar with technology or who receive social support; in some cases, the favorable assessments were directed not at the entire system, but rather at specific features that participants found useful. This suggests that while the system offers clear advantages for some users, further efforts are needed to ensure its accessibility and inclusiveness for the broader older adult population (Almulhem 2023).

Overall, our findings indicate a sequential yet cyclical process in older adults’ interaction with digital health technologies. Emotional perceptions (“We have grown old…”) shape cognitive appraisals (“It is necessary to be prepared for everything”), and those appraisals, in turn, drive digitalhealth behavior toward one of two poles—avoidance (“It is difficult to keep up with the digital age”) or engagement (“It is not difficult”). Positive experiences can reverse this cycle by boosting emotional confidence and lowering the cognitive threshold for use. The model therefore suggests that training interventions should begin by addressing the emotional layer, while design improvements should focus on reducing cognitive load.

### 4.5 Implications for Public Health Nursing

Public health nurses play a critical role in improving digital health literacy among older adults (Haupeltshofer et al. 2020). To support this, small‐group, hands‐on training sessions should be organized in community settings such as family health centers, senior activity centers, or municipal community hubs. These sessions should focus on practical skills, including how to access and navigate commonly used platforms such as e‐Nabız and MHRS for scheduling appointments, accessing test results, and communicating with healthcare providers.

Training programs should be tailored to the digital literacy levels, cognitive capacities, and device accessibility of older individuals. When necessary, one‐on‐one guidance can be provided by nurses or other primary care staff. Simple user guides and visual aids designed to address common challenges can also be distributed to reinforce learning (Almulhem 2023; World Bank 2023).

In addition, nurses can act as advocates by collecting feedback from older adults and sharing recommendations with system designers and policymakers (Fitzpatrick 2023). These recommendations may include incorporating larger font sizes, voice‐guided instructions, and simplified menu structures to make digital health platforms more age‐friendly.

Furthermore, public health nurses can collaborate with local leaders such as neighborhood heads, religious leaders, and non‐governmental organizations that serve older populations (Frishammar et al. 2023). Together, they can organize awareness sessions, mobile outreach days, and home visits to provide personalized digital health support.

Implementing these strategies will enhance older adults’ access to healthcare services, reduce digital disparities, and improve the overall efficiency and inclusiveness of health systems (Kaihlanen et al. 2022).

### 4.6 Limitations and Future Research

The findings may not fully reflect the perceptions and experiences related to all digital health systems, as they focus on digital health information systems more frequently used in Türkiye. Additionally, the results represent a relatively small group of older adults, whose perceptions are shaped by specific socioeconomic, social, cultural, and political factors unique to this population. Although the sample size was relatively small, it aligns with the goals of qualitative research, which prioritizes depth over breadth. Nevertheless, the limited number of participants may affect the transferability of the findings to broader populations. Moreover, the sample predominantly consisted of male participants and did not include university graduates, factors which may affect the generalizability of the results. However, considering the relatively low university graduation rates among older adults in Türkiye, this limitation reflects broader demographic characteristics. The translation of interview texts from Turkish to English presents another limitation, as some culture‐specific concepts may have been altered during the translation process.

Future studies should broaden the scope of investigation into older adults’ access to and utilization of digital health systems. Comparative research across different geographical, cultural, and socioeconomic contexts can help identify common challenges and tailor specific solutions to address them. Intervention studies evaluating the effectiveness of digital literacy programs can provide evidence‐based strategies to make digital health systems more inclusive for older adults. Longitudinal research could explore changes in older adults' knowledge and usage patterns of digital health systems over time. Furthermore, studies focusing on older adults from low‐income or marginalized groups are crucial for identifying barriers to equitable access and developing strategies to overcome them.

## 5 Conclusion

This study, which explores the perceptions and experiences of older adults in Türkiye regarding widely used digital health information systems (e‐Pulse and the CPAS), reveals several key findings. Older adults generally prefer traditional methods when seeking healthcare services and encounter barriers such as lack of knowledge, difficulty of use, and technological anxiety when engaging with digital health systems. While some participants expressed anxiety and discomfort due to the perceived difficulty of using digital health applications, others indicated that they did not find such applications challenging and felt competent in their usage.

These findings highlight critical considerations for public health policy. First, the ongoing preference for traditional healthcare suggests the value of hybrid service delivery models that combine digital tools with in‐person options. Second, the widespread lack of digital skills points to the necessity of implementing age‐sensitive digital literacy programs within primary healthcare and community settings. Third, the perceived complexity of existing systems underscores the need for national usability standards that promote simple, accessible, and age‐friendly platform design.

Furthermore, the influence of social support on user confidence suggests that community‐based strategies—such as involving local leaders or trained digital ambassadors—can play a vital role in promoting adoption. Addressing emotional and cognitive barriers through guided assistance and inclusive outreach is essential to ensure that digital transformation in healthcare does not widen health disparities among older adults.

## Conflicts of Interest

The authors declare no conflicts of interest.

## Data Availability

The data generated and analyzed in this study are not publicly available due to privacy and ethical considerations. However, the data can be provided upon reasonable request from the corresponding author.
